# Social Service Department at the General Hospital

**Published:** 1911-02

**Authors:** 


### BUFFALO MEDICAL JOURNAL

**A Monthly Review of Medicine and Surgery**

*EDITOR*

WILLIAM WARREN POTTER, M. D.

All communications, whether of a literary or business nature, books for review and exchanges, should be addressed to the editor, 238 DELAWARE AVENUE, BUFFALO, N.Y.

VOL. LXVI. FEBRUARY, 1911. No. 7

### Social Service Department at the General Hospital.

WE made reference in the January edition of the JOURNAL to the establishment of a social service department at the Buffalo General Hospital, which had been placed in charge of Miss Lucy Stockton. This new bureau was opened January 3, 1911, with Miss Stockton in charge assisted by Miss Grace Menzie, one of the district nurses. Miss Stockton, daughter of Dr. Charles G. Stockton, and Miss Menzie have spent sometime in an investigation and study of this important work in Boston, particularly as carried on at the Massachusetts General Hospital.

One of the important features of the work is to provide temporary homes for convalescents until they become hardy enough to return to work. Nothing is sadder than to see a hospital patient turned out into the world after illness, without strength, or food, or clothing—without employment or ability to work if employment were offered. But the convalescent must make way in spite of this hardship for the incoming patient with acute disease. And so Miss Stockton and her coadjutors will relieve these poor feeble men and women from the great burden carried between the sick bed and complete recovery, a period always trying in the extreme, but a hundred fold more so in the face of poverty and want.

After a study of the situation and when other needs become manifest, these will be added to the duties of the social service administration. The district nursing association will coöperate in this important work, one of the principal features of which it is presumed will be, securing care for children during the convalescence of parents. No doubt, too, there will be instruction in personal and home hygiene, giving aid to those needing and seeking employment, besides the furtherance of fresh air work, and many other useful, philanthropic, and even charitable acts toward those in need physically, mentally, and morally. We cannot follow out the various ramifications of this important work; they are too numerous, too important, ever increasing in influence, uplifting in effect, and exalting in action to present in a single article, having for its only object only a casual reference to the importance of the subject and the general features involved.

To accomplish the purposes aimed at by these philanthropic and self-sacrificing young women, will require the coöperation of physicians, nurses, and philanthropic persons in general, as well as courts and court officers and almost every useful citizen. The benevolent nature of this work should appeal to the better heart and conscience of all the people. Men and women in all the walks of life should make haste to offer their best aid in perfecting the scheme and making it effective in all its details.

We offer our congratulations to Miss Stockton and her associates in the work so auspiciously begun and trust a full measure of success may be meted out to them.

